# Too few, too late: U.S. Endangered Species Act undermined by inaction and inadequate funding

**DOI:** 10.1371/journal.pone.0275322

**Published:** 2022-10-12

**Authors:** Erich K. Eberhard, David S. Wilcove, Andrew P. Dobson

**Affiliations:** 1 Department of Ecology, Evolution, and Environmental Biology, Columbia University, New York, New York, United States of America; 2 Department of Ecology and Evolutionary Biology, Princeton University, Princeton, New Jersey, United States of America; 3 Princeton School of Public and International Affairs, Princeton University, Princeton, New Jersey, United States of America; 4 Santa Fe Institute, Santa Fe, New Mexico, United States of America; University of Bucharest, ROMANIA

## Abstract

This year, the Conference of Parties to the Convention on Biological Diversity will meet to finalize a post 2020-framework for biodiversity conservation, necessitating critical analysis of current barriers to conservation success. Here, we tackle one of the enduring puzzles about the U.S. Endangered Species Act, often considered a model for endangered species protection globally: Why have so few species been successfully recovered? For the period of 1992–2020, we analyzed trends in the population sizes of species of concern, trends in the time between when species are first petitioned for listing and when they actually receive protection, and trends in funding for the listing and recovery of imperiled species. We find that small population sizes at time of listing, coupled with delayed protection and insufficient funding, continue to undermine one of the world’s strongest laws for protecting biodiversity.

## Introduction

Accelerating rates of species extinction are a matter of global concern [1] as exemplified in the Intergovernmental Science-Policy Platform on Biodiversity and Ecosystem Services (IPBES) report that predicted the loss of over 1 million species in the foreseeable future, which will also have significant impacts on the delivery of ecosystem services [2]. The prevention of species extinction is a primary goal of the Convention on Biological Diversity and the UN Sustainable Development Goals. In the United States, the strongest law to prevent species extinctions is the Endangered Species Act (ESA) [3], which has served as a model for other nations since its passage by the Nixon Administration in 1973. A longstanding concern of both supporters and opponents of the law has been the relatively low number of listed species that have successfully recovered to the point where they no longer need protection. In the 48 years since enactment of the ESA, only 54 US species have been declared fully recovered and delisted [4].

Multiple explanations have been given for this low rate of recovery including: (a) a pattern of not protecting species until their populations have reached very low levels, which increases both the time to recovery and the likelihood that species will vanish entirely due to environmental, genetic, and demographic stochasticity [5]; (b) a lack of incentives to landowners to participate actively in efforts to increase populations of endangered species [6]; and (c) inadequate funding for recovery actions [7]. Here, we have used data from the *Federal Register* to examine trends in the population sizes of species at time of listing and the levels of funding available to list and recover them.

Evidence that species are not being protected under the ESA until their populations have reached dangerously low levels was initially provided in a 1993 paper by Wilcove et al. [8]. The authors found that the median population size at time of listing during the second decade of ‘legal protection’ by the ESA (1985–1991) was just 1075 for vertebrates and 999 individuals for invertebrates. The median population size at listing for plant species was less than 120 individuals. We repeated their methodology to determine whether the US Fish & Wildlife Service (FWS) has become more proactive as we approach the 50^th^ anniversary of the ESA and roughly 30 years since attention was first drawn to this problem.

We also examined trends in the length of time between when a species is identified as potentially deserving of protection and when it actually receives that protection under the ESA (hereafter, “wait times”). It should be noted that, in recent years, most of the species added to the ESA have been the result of petitions from non-governmental entities to FWS requesting protection of a given species. Frequently, listing follows litigation brought by environmental organizations when petition decisions are overdue or petitions are denied [9].

Finally, we examined trends in funding for the listing and recovery of imperiled species (we use “imperiled” to include both Endangered and Threatened species protected under the ESA, and, unless indicated otherwise, we use the word “species” to refer to any entity protected under the ESA, including subspecies and vertebrate populations). We give particular attention to trends in funding *per species*, in order to account for changes in the number of species listed each year.

## Materials and methods

The list of plants and animals granted protection under the ESA was collated from annual listing records available through the U.S. Fish & Wildlife Service’s Environmental Conservation Online System (ECOS). Population data were obtained from Final and Proposed Listing Notices issued by the U.S. Fish & Wildlife Service. Our analysis was restricted to wild populations of plants and animals known to occur in the United States and its territories and did not include captive populations.

When presented with a range of values, or an upper limit, for the total number of individuals or populations at time of listing, we favored interpretations that maximized population size. For example, if a population was said to be “between 500 and 1000” individuals, we recorded the population as being 1000 individuals at time of listing. Similarly, a population said to be “<1000” was recorded as being 999 individuals at time of listing. This was done in order to obtain the largest possible estimate of each plant and animal population at time of listing, making our subsequent analyses an optimistic “best case scenario”. Six species were listed with no known individuals or populations surviving in the wild. In these instances, the total number of individuals or total number of populations was recorded as being zero. Population data for plants and animals listed between 1985–1991 were obtained from Wilcove et al. in order to facilitate comparison with their results. We performed a non-parametric Wilcoxon Rank-sum Test to compare the medians of continuous variable *x*, the number of individuals at time of listing for species listed between 1985–1992, and continuous variable *y*, the number of individuals at time of listing for species listed between 1993–2020. The same approach was used to compare the median number of populations at time of listing for each time period. We adopted a significance threshold of *p* = 0.05.

Data for Resource Management Appropriations (discretionary funding that supports the management and recovery of imperiled species by FWS) and Section 4 Appropriations (funding allocated specifically to ESA listing activities) were obtained from the text of annual federal budget legislation and corrected for inflation to 2019 USD. These documents are publicly accessible through the *Federal Registrar*. Funding per species was defined as the average funding available for the management of each species in a given year. We calculated this value by dividing annual Resource Management Appropriations by the total number of species protected under the ESA as of the first day of that calendar year.

## Results

**Fig 1:**
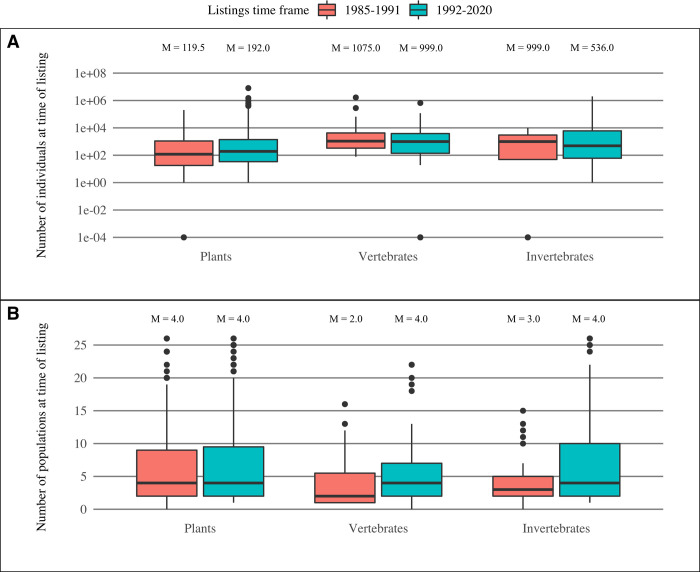
Species status at time of listing, 1985–1991 versus 1992–2020. (A) Comparison of population size at time of listing for plants and animals. There are no significant differences between the two periods (Wilcox Test values of p = 0.08, p = 0.41, and p = 0.66, for plants, vertebrates and invertebrates respectively). (B) Comparison of number of populations at time of listing. There are no significant differences between the two periods for plants and invertebrates. Values of zero indicate species for which there were either no known individuals or no known populations at time of listing. Median values shown above each plot.

From 1992–2020, the FWS listed a total of 970 species for protection under the ESA; 68% of these listings were plants, 18% were invertebrates, and 14% were vertebrates. Full species accounted for the majority of listings during this period (80%). Of the species listed, 602 had data on their total population size (total number of individuals) at time of listing, and 843 had data on the number of populations at time of listing. For each taxonomic group analyzed, the total population size at time of listing (Fig 1A) did not differ significantly between the 1985–1991 and 1992–2020 time periods (Wilcox Test values of p = 0.08, p = 0.41, and p = 0.66, for plants, vertebrates and invertebrates respectively). For plants and invertebrates, the total number of populations at time of listing (Fig 1B) also did not differ significantly between the 1985–1991 and 1992–2020 time periods (p = 0.91 and p = 0.06, respectively). However, the median number of vertebrate populations at time of listing was slightly greater in the 1992–2020 time period, increasing from 2 to 4 populations (p = 0.04).

Our analysis revealed longer wait times for species petitioned for listing during the 2000–2009 period (median = 9.1 years), compared to those petitioned for listing during the 1992–1999 period (median = 5.9 years), followed by shorter wait times for species petitioned for listing during the 2010–2020 period (median 3.0 years). The number of petitions received during each period also varied greatly (*n* = 49, 203 and 26 for 1992–1999, 2000–2009 and 2010–2020, respectively). While wait times seem to decrease when fewer species are listed, there are insufficient data to test whether this effect is significant.

**Fig 2:**
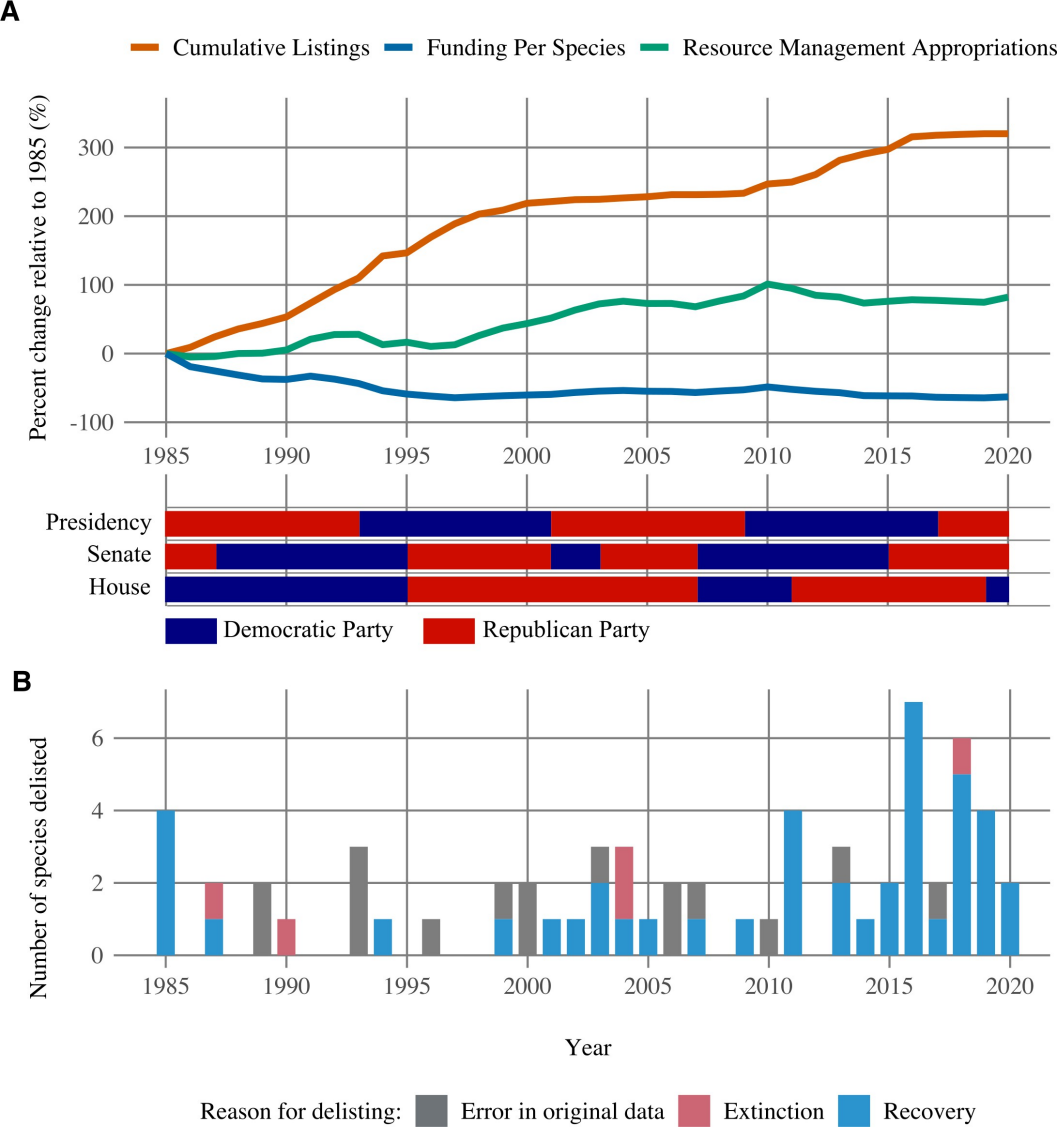
Trends in number of species protected under the ESA and funding for species management, 1985–2020. (A) Change in cumulative number of ESA listings compared to change in Resource Management Appropriations. The lower timeline illustrates political control of the Presidency and, by a majority, each house of Congress. (B) Number of species delisted for various reasons.

Resource Management Appropriations climbed modestly from 1996–2010 before beginning a decade-long decline that was halted only in 2020 (Fig 2A). The same trend is observed in Section 4 Appropriations, which peaked in 2010 at $25.9 million USD before dropping to $20.1 million USD by 2020. Concurrently, the number of species listed for protection under the ESA increased by over 300% between 1985–2020. As such, Resource Management Appropriations, when measured on a *per species* basis, have dropped by nearly 50% since 1985.

## Discussion

Our analysis of trends in the protection of imperiled species under the US Endangered Species Act warrants a limited amount of optimism and a larger amount of pessimism: Most species are not receiving protection until they have reached dangerously low population sizes. First reported in 1993, this pattern has persisted throughout the intervening quarter century. We suspect that most of the species listed since 1993 had fallen to low population levels well before the time span of our study, a reflection of past anthropogenic activities. Their protection under the ESA implies a painfully slow process of clearing a backlog of rare but unprotected species as opposed to a failure to respond to recent, rapid population declines in formerly more common species.

The wait-times between when a species is first petitioned for protection under the ESA and when it finally receives that protection have waxed and waned since 1992. The period with the longest median wait time (2000–2009, with a median wait-time of 9.1 years), was also the period when the greatest number of petitions were received by FWS (*n* = 203). The period with the shortest median wait time (2010–2020 with a median wait-time of 3.0 years) was the period when the fewest number of petitions were received (*n* = 26). This suggests that wait times may be exacerbated when limited resources for listing are strained by a large influx of petitions. Consistently, very few species have received protection in the two-year period that is prescribed in the ESA. For species with very small or rapidly declining populations, a multi-year delay in receiving protection increases the risk of extinction.

Our data suggest that inadequate funding has persisted for decades, with no clear relationship as to which political party is in power (Fig 2A). The unfortunate conclusion is that FWS is being asked to do more with less resources. The combination of delays in listing rare species, the typically mall population sizes of species at time of listing, and inadequate funding for recovery actions, are the key factors that can explain the relatively small number of listed species that have fully recovered (Fig 2B). Resource allocation frameworks and other decision-support tools can help FWS make the most efficient use of the funds it receives [10], but increased funding is essential for sustained, substantial progress in protecting imperiled species [11, 12]. Studies have shown that government expenditures for imperiled species management do contribute to an improvement in recovery status and averted extinctions [13].

Although the US is one of only a handful of nations that have failed to ratify the Convention on Biological Diversity, its commitment to preventing the loss of its own “non-voting” species dates back nearly half a century to the passage of the ESA in 1973. In December 2022, when international leaders gather in Montréal, Canada for the 15^th^ meeting of the Conference of Parties to the Convention on Biological Diversity, the failure of the US to have solved the funding gaps that hamper the ESA will stand as a stark reminder of the difference between a visionary promise and its functional implementation.

## Supporting information

S1 Data (XLSX)
